# Carboxyhemoglobin Does Not Predict the Need of Mechanical Ventilation and Prognosis during COPD Exacerbation

**DOI:** 10.1155/2022/6689805

**Published:** 2022-04-16

**Authors:** Shimon Izhakian, Eitan Harper, Oleg Gorelik, Assaf Frajman, Ori Mekiten, Adina Bar-Chaim, Mordechai Reuven Kramer

**Affiliations:** ^1^Department of Internal Medicine F, Yitzhak Shamir Medical Center, Zerifin 7033001, Israel; ^2^Affiliated to Sackler Faculty of Medicine, Tel Aviv University, Ramat Aviv, Israel; ^3^Rabin Medical Center, Pulmonary Institute, Petah Tikva, Israel; ^4^Division of Clinical Laboratories, Yitzhak Shamir Medical Center, Zerifin 7033001, Israel

## Abstract

**Background:**

Carboxyhemoglobin (COHb) is a complex formed by the binding of carbon monoxide to hemoglobin in blood. Higher COHb levels have been associated with poor prognosis in a variety of pulmonary disorders. However, little is known regarding the prognostic significance of COHb among individuals with chronic obstructive pulmonary disease (COPD) exacerbation.

**Methods:**

In a retrospective study, we evaluated associations of venous COHb levels on hospital admission with the need for invasive mechanical ventilation, in-hospital mortality, and rehospitalization, among 300 patients hospitalized for COPD exacerbation in internal medical wards.

**Results:**

Rates of in-hospital death and 1-year recurrent hospitalizations were 11.0% and 59.6%, respectively. COHb levels were not significantly associated with in-hospital mortality (OR = 0.82, *P*=0.25, 95% CI 0.59–1.15) or with 1-year rehospitalizations (OR = 0.91, *P*=0.18, 95% CI 0.79–1.04). The mean COHb level did not differ significantly between patients who needed invasive mechanical ventilation and those who were not invasively mechanically ventilated during the current hospitalization (2.01 ± 1.42% vs. 2.19 ± 1.68%, *P*=0.49).

**Conclusions:**

Among patients hospitalized with COPD exacerbation in internal medicine wards, COHb levels on admission were not associated with invasive mechanical ventilation treatment, rehospitalizations, or mortality.

## 1. Introduction

Carboxyhemoglobin (COHb) is the product of the reaction between carbon monoxide and hemoglobin. The sources of carbon monoxide are the endogenous metabolism and exogenous production in the environment. Endogenously produced carbon monoxide is not only toxic waste but is also involved in many physiological functions such as respiratory regulation [1], neuronal signaling [2], blood pressure regulation [3], inflammatory response, angiogenesis, and vascular remodeling [4].

In the last decade, evidence has accumulated regarding an association between higher COHb levels and poor prognosis in various disorders such as pneumonia [5], pulmonary embolism [6], sepsis [7], postresuscitation care [8], and critical illness [9]. Increased concentrations of COHb were also found to be associated with pulmonary inflammation among patients with asthma [10] and interstitial lung disease [11].

Information regarding the clinical significance of COHb levels in patients with chronic obstructive pulmonary disease (COPD) is limited [12–15]. Increased COHb levels have been strongly correlated with pulmonary function [12, 13], Global Initiative for Chronic Obstructive Lung Disease (GOLD) stage [12, 13], and C-reactive protein (CRP) values [12] during COPD exacerbation. Moreover, higher COHb concentrations have been associated with mortality among persons with COPD in general patient populations [14, 15]. However, no study has explored the utility of COHb in predicting the need of mechanical ventilation, rehospitalization, and in-hospital mortality during hospitalization with COPD exacerbation.

We hypothesized that, among patients hospitalized for COPD exacerbation, higher COHb levels may be associated with poor prognosis. Thus, the aim of this study was to evaluate associations of COHb concentrations at hospital admission with invasive mechanical ventilation treatment, mortality, and recurrent hospitalizations, among patients hospitalized for exacerbated COPD in internal medicine wards.

## 2. Methods

### 2.1. Study Population and Design

This retrospective observational single-center study was conducted in Yitzhak Shamir (Assaf Harofeh) Medical Center, a tertiary care university hospital located at Zerifin, Israel. Study inclusion criteria were as follows: admission from the emergency department at one of seven internal medicine departments (250 beds in total) during January–June 2013 for COPD exacerbation, a documented diagnosis of COPD by spirometry and clinical symptoms, the availability of a blood gas measurement within the first 24 hours of hospitalization, and age ≥18 years.

Exclusion criteria were admission from the emergency department directly to intensive care units and the probability of a diagnosis other than COPD. The study was carried out in accordance with the Declaration of Helsinki and was approved by the institutional ethics committee.

### 2.2. Definition of COPD and Exacerbation

We defined patients as having COPD according to GOLD guidelines 2020. This diagnosis entails respiratory symptoms and airflow limitation due to airway or alveolar abnormalities, which are usually caused by significant exposure to noxious particles or gases. Spirometry was required for making the diagnosis, with the presence of postbronchodilators FEV1/FVC below 0.7. Spirometry was performed by Zan-530 plethysmography (nSpire Health, Germany), according to the updated guidelines of the European Community for Steel and Coal (ECSC) and the European Respiratory Society (ERS) statement [16]. COPD exacerbation was defined as worsening of baseline symptoms such as cough, dyspnea, or sputum production.

### 2.3. Data Collection

**Figure 1:**
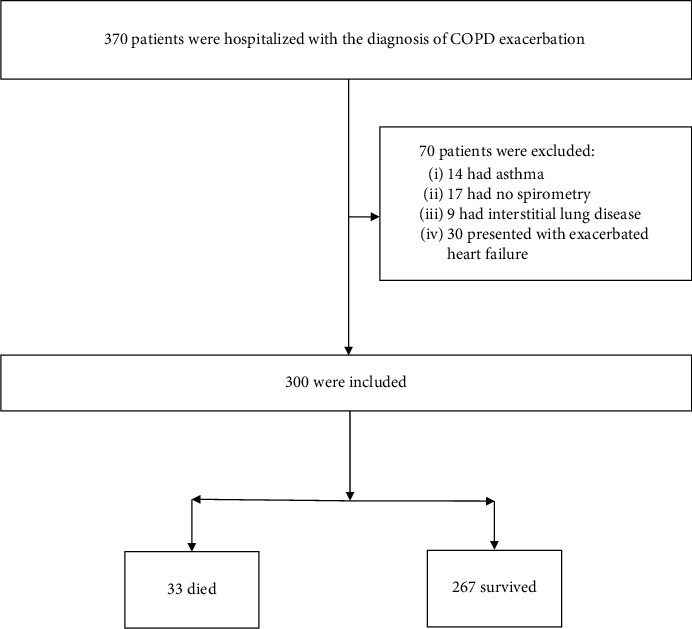
Flowchart presenting the study design.

Data were obtained from electronic medical record database systems that integrate medical information from all hospitals in Israel. We identified 370 patient charts according to the abovementioned eligibility criteria. We further excluded 70 patients due to probable misdiagnosis (14 had asthma, 17 had no documented diagnosis of COPD by spirometry, 9 had interstitial lung disease, and 30 presented with exacerbated heart failure (Figure 1)).

The collected data included the following variables: age, gender, smoking status, chronic disorders, length of hospital stay, treatment with invasive mechanical ventilation, in-hospital death, and rates of in-hospital mortality and rehospitalizations at 90 days, 6 months, and one year from the first admission. We double checked the information regarding in and out-hospital mortality, based on the registry of the Ministry of Internal Affairs. We also collected data regarding values of COHb (as a percentage of total hemoglobin) measured at the first day of admission and blood oxygen saturation. COHb was measured spectrophotometrically using a blood gas analyzer (Roche Omni S).

### 2.4. Statistical Analysis

Descriptive data were expressed as means and standard deviations (SDs) and as numbers and percentages of presenting patients. We used chi-square tests for categorical variables and Student's *t*-test for continuous variables. *P* values of <0.05 were considered significant. Variables that were found to be associated with mortality and rehospitalization on univariate analysis, COHb levels, and possible confounders were reevaluated by the stepwise logistic regression model. Spearman's correlation coefficient was calculated between COHb levels and the length of hospitalization. The data were assembled using Excel software, and statistical analysis was performed using the SAS software version 9.2 (SAS Institute Inc., Cary, NC, USA).

## 3. Results

Of the 300 patients who were hospitalized due to COPD exacerbation, 267 survived the first hospitalization, while 33 (11.0%) died. Compared to those who survived, patients who died during the first hospitalization were older and were more likely active smokers, with lower oxygen saturation at admission (Table 1). The proportions of patients with comorbidities did not differ significantly between the groups (Table 1). During the first admission, 49 patients (16.3%) were treated with invasive mechanical ventilation.

The rates of recurrent hospitalizations within 90 days, 6 months, and one year following the first admission were 41.0%, 49.6%, and 59.6%, respectively. The respective 90-day, 6-month, and 1-year mortality rates were 20.6%, 21.6%, and 28.6%.

The mean COHb level in blood was higher among active than nonactive smokers (Table 2). Mean COHb values were not found to be associated with treatment with invasive mechanical ventilation, recurrent hospitalizations, or mortality (Table 2). Higher COHb levels were not found to be correlated with longer hospitalization (*R* = 0.01).

On multivariate analysis, the following variables were most significantly associated with in-hospital mortality: older age (OR = 1.06, *P* < 0.001, 95% CI 1.02–1.10), lower oxygen saturation levels (OR = 1.07, *P*=0.002, 95% CI 1.02–1.11), active smoking status (OR = 2.38, *P*=0.4, 95% CI 1.01–5.58), home oxygen therapy prior to admission (OR = 2.92, *P*=0.3, 95% CI 1.10–7.74), and home treatment with noninvasive mechanical ventilation prior to admission (OR = 2.97, *P*=0.02, 95% CI 1.17–7.54). In this analysis, COHb levels did not predict in-hospital mortality (OR = 0.82, *P*=0.25, 95% CI 0.59–1.15). Moreover, COHb concentration was not associated with mortality and rehospitalization within 90 days (OR = 0.90, *P*=0.32, 95% CI 0.73–1.10 and OR = 0.86, *P*=0.08, 95% CI 0.73–1.02, respectively), 6 months (OR = 0.89, *P*=0.27, 95% CI 0.72–1.09 and OR = 0.93, *P*=0.36, 95% CI 0.81–1.07, respectively), and one year (OR = 0.88, *P*=0.17, 95% CI 0.73–1.05 and OR = 0.91, *P*=0.18, 95% CI 0.79–1.04, respectively) of the first admission for COPD exacerbation.

## 4. Discussion

In this study, we explored associations of the COHb level with the need for invasive mechanical ventilation, rehospitalizations, and mortality among patients hospitalized for COPD exacerbation in internal medicine wards. We found that COHb levels at hospital admission did not predict in-hospital death. To our best knowledge, this is the first study that explored the utility of COHb as a prognostic factor in acute exacerbation in the setting of an internal medicine ward. Our results are consistent with those of Fazekas et al. who measured arterial COHb of 868 patients in the ICU and concluded that it does not qualify as a predictive marker for ICU mortality [17]. In contrast to our findings, in two studies that were conducted in general patient populations in Scotland [14] and London [15], as periodic health examinations for men and women aged 35–64 years, associations of higher COHb levels with mortality were reported among individuals with COPD comorbidity. The results of our multivariate analysis provide a possible explanation for the lack of a significant association of COHb with mortality in our study. Accordingly, among patients hospitalized for exacerbated COPD, variables other than COHb, such as older age, lower level of oxygen saturation, active smoking, and home treatment with oxygen and noninvasive mechanical ventilation, were most significantly associated with poor prognosis. Surprisingly, in contrast to data from the literature [18], none of the evaluated comorbidities (hypertension, hyperlipidemia, diabetes mellitus, heart failure, ischemic heart disease, chronic renal failure, and cerebrovascular disease) was found to be significantly associated with mortality. These findings may be explained by the relatively small sample size. Moreover, the lack of collected data regarding a number of relevant variables precluded evaluating the prognostic significance of any comorbidity index that has been reported in association with mortality among patients with COPD [18].

Another interesting observation in the current study is the lack of significant associations of COHb levels with rates of rehospitalization or with treatment with invasive mechanical ventilation. To the best of our knowledge, evaluation of these associations in patients with COPD has not been reported in the medical literature. In two studies on patients with COPD exacerbation, associations were reported of higher COHb levels with severity of COPD according to the GOLD stage [12, 13]. Yasuda et al. also reported an inverse correlation of arterial COHb levels with arterial blood partial oxygen pressure and a positive correlation with serum CRP values [12]. It has been suggested that endogenous carbon monoxide is endogenously produced by the action of heme oxygenase 1 (HO-1). This enzyme is induced in response to hypoxia, inflammation, and oxidative stress [19]. It has also been postulated that COHb concentration might increase through the production of proinflammatory cytokines and nitric oxide in the airway due to viral infections [20] and through reactive oxygen species from neutrophils in bacterial infection [21]. We did not collect data regarding GOLD staging and inflammatory markers. Moreover, we evaluated venous COHb level, which is known to be highly correlated with arterial COHb [22]. Due to the retrospective design of this study, we did not examine the relations of timing and amount of cigarette smoking prior to admission to COHb level. Notably, previous studies in cohorts of people who smoked showed that COHb concentration did not increase during the day, when cigarettes were smoked, but rather remained stable for each person [23].

Our study has a number of limitations. First, this was a single-center study and the results may not be generalizable to other medical centers. Second, the relatively small sample may have affected statistical power for comparison of some relevant data. Third, due to the retrospective design, missing data may have affected the results. Finally, the relation between standard comorbidity indexes and prognosis was not assessed. The strength of our study is the evaluation of associations of COHb with various previously unexamined outcomes among patients hospitalized for COPD exacerbation.

## 5. Conclusions

Among patients admitted with COPD exacerbation to internal medical wards, COHb levels were not significantly associated with invasive mechanical ventilation treatment, rehospitalizations, and mortality. These results are preliminary, and future prospective studies will be useful for better understanding the role of the COHb as a prognostic biomarker of COPD exacerbation.

## Figures and Tables

**Table 1:** Baseline characteristics of patients with chronic obstructive pulmonary disease (COPD) according to survival during hospitalization for exacerbated COPD.

Variable	Survived	Did not survive	*P* value
	*n* = 267	*n* = 33	

Age, y	72.9 ± 12.7	80.6 ± 10.8	0.001>
Male sex	133 (49.8%)	22 (66.6%)	0.07
Active smoking	159 (59.5%)	26 (78.7%)	0.04
Hypertension	184 (68.9%)	22 (66.6%)	0.75
Hyperlipidemia	125 (46.8%)	17 (51.2%)	0.61
Diabetes mellitus	107 (40%)	12 (36.6%)	0.70
Heart failure	81 (30.3%)	11 (33.3%)	0.68
Ischemic heart disease	74 (27.7%)	11 (33.3%)	0.46
Chronic renal failure	49 (18.3%)	8 (24.2%)	0.36
Cerebrovascular disease	32 (11.9%)	7 (21.2%)	0.11
Home oxygen therapy	100 (37.4%)	13 (39.3%)	0.03
Home noninvasive mechanical ventilation therapy	56 (20.9%)	9 (27.2%)	0.02
Blood oxygen saturation on admission (%)	89.4 ± 8.1	83.1 ± 9.9	0.002

Data are expressed as means ± SD or numbers (percentages) of presented cases.

**Table 2:** Association of COHb levels with smoking and outcomes.

Variable	Mean ± SD COHb (%)	*P* value

Active smoking		
Yes (*n* = 185)/no (*n* = 111)	3.16 ± 2.38/1.61 ± 0.52	<0.001
Treatment with invasive mechanical ventilation during the current hospitalization		
Yes (*n* = 49)/no (*n* = 251)	2.01 ± 1.42/2.19 ± 1.68	0.49
In-hospital mortality		
Yes (*n* = 33)/no (*n* = 267)	1.85 ± 0.75/2.20 ± 1.72	0.25
90 days rehospitalization		
Yes (*n* = 123)/no (*n* = 177)	1.96 ± 1.16/2.30 ± 1.90	0.08
6 months rehospitalization		
Yes (*n* = 149)/no (*n* = 151)	2.07 ± 1.32/2.24 ± 1.90	0.36
1 year rehospitalization		
Yes (*n* = 179)/no (*n* = 121)	2.05 ± 1.28/2.31 ± 2.06	0.18

Data are expressed as means ± SD or numbers of presented cases. COHb, carboxyhemoglobin.

## Data Availability

The data used to support the findings of this study are available from the corresponding author upon request.
